# Optimization, purification, and characterization of xylanase production by a newly isolated *Trichoderma harzianum* strain by a two-step statistical experimental design strategy

**DOI:** 10.1038/s41598-022-22723-x

**Published:** 2022-10-22

**Authors:** Priyashini Dhaver, Brett Pletschke, Bruce Sithole, Roshini Govinden

**Affiliations:** 1 Discipline of Microbiology, School of Life Sciences, Westville Campus, University of KwaZulu-Natal, Durban, 4000 South Africa; 2 Enzyme Science Programme (ESP), Department of Biochemistry and Microbiology, Rhodes University, Makhanda (Grahamstown), Eastern Cape South Africa 6139; 3 Biorefinery Industry Development Facility, Council for Scientific and Industrial Research, Durban, 4000 South Africa; 4 Discipline of Chemical Engineering, University of KwaZulu-Natal, Durban, 4000 South Africa

**Keywords:** Biotechnology, Microbiology

## Abstract

Xylanases are hydrolytic enzymes with a wide range of applications in several industries such as biofuels, paper and pulp, food, and feed. The objective of this study was to optimize the culture conditions and medium components for maximal xylanase production from a newly isolated *Trichoderma harzianum* strain using the Plackett–Burman Design (PBD) and Box Behnken Design (BBD) experimental strategies. Xylanase production was enhanced 4.16-fold to 153.80 U/ml by BBD compared to a preliminary one-factor-at-a-time (OFAT) activity of 37.01 U/ml and 2.24-fold compared to the PBD (68.70 U/ml). The optimal conditions for xylanase production were: 6 days of fermentation, incubation temperature of 70 °C, pH 5.0, agitation of 160 rpm, and 1.2% wheat bran and ammonium sulphate. The experimental design effectively provided conditions for the production of an acidic-thermostable enzyme with exciting potential for application in animal feed improvement. The acidic-thermostable xylanase was purified from the submerged culture and SDS-PAGE analysis revealed a molecular weight of 72 kDa. This protein had maximum xylanolytic activity at pH 6.0 and 65 °C and was stable for 4 h retaining > 70% activity and exhibited substrate specificity for beechwood xylan with a *K*_*m*_ of 5.56 mg/ml and *V*_*max*_ of 1052.63 µmol/min/mg. Enzyme activity was enhanced by Fe^2+^, Mg^2+^, and Zn^2+^. There was an absence of strong inhibitors of xylanase activity. Overall, these characteristics indicate the potential for at least two industrial applications.

## Introduction

After cellulose, hemicellulose is the second most abundant terrestrial polysaccharide composed of ß-1,4-d-xylopyranoside residues and ß-1,4-xylan as main constituents with arabinosyl and acetyl side chains^1^. Hemicellulose is a short crosslinked polymer compared to cellulose, which is a long straight-chain homopolymer. Xylans have a β-(1,4) linked backbone made of d-xylose and there are three subtypes of xylan based on the side chain. The subtypes are homoxylan, glucuronoxylan, and arabinoxylan. Homoxylan is only found in two or three types of plants and is mostly cross-linked by β-(1,4)-glycosidic bonds. Xylan is a renewable biomass resource that has potential as a substrate in many production processes. However, it must be hydrolysed to xylose and xylo-oligosaccharides which can be accomplished by xylanolytic enzymes. Among them, xylanases deserve special attention as they degrade the major hemicellulosic polysaccharides by catalyzing the hydrolysis of xylopyranosyl linkages of ß-1,4-xylan^2^. The main enzymes involved are endo-1,4-β-xylanases which make random cuts in the xylan backbone and β-xylosidases which are exoglycosidases with the ability to degrade the non-reducing ends of xylooligosaccharides into xylose. The side groups in xylans are cleaved by α-l-arabinofuranosidases (EC 3.2.1.55), α-glucuronidases (EC 3.2.1.139), and acetyl xylan esterases (EC 3.1.1.72)^3^. The most common microbial xylanases that would hydrolyze all types of xylan are grouped based on amino acid similarities and structural characteristics, into glycoside hydrolase (GH) families 10 or 11 in the Carbohydrate-Active enzyme (CAZy) database (http://www.cazy.org)^4^. GH10 families are able to catalyze the hydrolysis of a wide range of xylans, while GH11 families are known to cleave unsubstituted regions of arabinoxylan^4^. However, both GH10 and GH11 xylanases have applications in various industries such as food and feed^5^, biofuel production^6^, paper and pulp^7^, and medical and pharmaceutical^8^. Xylanases are also found in GH families 30, 8, and 5. The complete degradation of heterogeneous xylan into simple sugars requires the synergistic action of several inducible hemicellulases^9^.

Microbes such as bacteria, fungi, and actinomycetes are ubiquitous in nature^10^ and several endogenous and exogenous microbial enzymes have been widely explored, resulting in a variety of microorganisms commonly regarded as the most significant and convenient producers of large quantities of enzymes in a short period on inexpensive feedstock. Xylanases are produced by microbial biosynthesis for industrial applications^11,12^. Thermophilic fungi in particular are promising candidates for biotechnological applications due to their strong ability to degrade plant polysaccharide components and their robustness under harsh environmental conditions^2^. Many of the filamentous fungi that have been studied produce several xylanases and have high xylan-degrading ability. *Trichoderma* sp. and *Aspergillus* sp. are most frequently employed for industrial applications^9^ including the bioconversion of plant biomass into animal feed^5^, plant fertilizers, and chemicals for the food industry^9^.

The production of enzymes is costly, thus, to meet industrial demand, a low-cost growth medium is required for microbial growth and enzyme production. There are two possible cultivation methods for microbial xylanase production: solid-state and submerged fermentation^13^. Submerged fermentation technology has the advantage of short production periods to achieve high yields at low costs. Both the nutrient medium composition and culture conditions have a strong influence on xylanase production. The physical and chemical factors known to influence xylanase production are temperature, pH, incubation period, carbon and nitrogen sources and concentration, and agitation speed^14^. Temperature effects on enzyme production are predominantly related to the growth of the organism (mesophilic, psychrophilic, or thermophilic). The pH is one of the most important factors governing microbial growth due to their sensitivity to the hydrogen ion concentration in the medium^15^. It is also key to enzyme activity as it can alter the ionic charges on the molecule, which could cause changes to the enzyme’s shape (they may denature), and that usually leads to a reduction or loss of the catalytic properties of the enzymes and cessation of metabolic activity^16^.

Supplementation of the growth medium with carbon and nitrogen sources usually increases enzyme production as this provides an enriched environment for microbial growth^17^. Therefore, screening of the most influential factors and optimization of the growth conditions are essential to ensure maximal enzyme production, potentially significantly reducing production costs for xylanases^18^*.* There are two approaches to optimize the fermentation process classical and statistical. The classical approach is based on the testing of “one-factor-at-a-time (OFAT)” and the statistical approach includes the Plackett–Burman design (PBD) and response surface methodologies (RSM).

The OFAT approach is a conventional single-dimensional investigation that involves changing one independent variable at a time while the others remain at their optimal level^19,20^. This is the main strategy used for selecting optimal conditions, which continues to be widely used in preliminary optimization studies^21^. The main disadvantages of OFAT are the partial explanation regarding the effect of the factors on the response and the absence of the interaction effects between the variables^22^. This method also involves a relatively high number of experiments, which makes it laborious and time-consuming^21^. Moreover, it may lead to unreliable results and inaccurate conclusions.

To resolve this problem, optimization studies can be carried out by using multivariate statistical methods^21^, PBD and RSM can potentially eliminate the limitations of the OFAT optimization process^23^. PBD is a powerful statistical technique for screening medium components in shake flask fermentation and reduces the total number of experiments^24^. This technique is useful and has been widely used as the first step of an optimization procedure, however, it cannot determine the interaction effects^23^ but allows the evaluation of the importance of each factor in moderately few experiments^25^. RSM using a Box–Behnken Design (BBD) is an effective optimization tool. The RSM design can provide the dependence of enzyme production on independent variables, predicted results for responses, and levels for independent variables in the form of mathematical models^26,27^.

Hydrolysis of xylan and hemicellulosic materials to various xylooligosaccharides has been accomplished using crude xylanases. However, to meet the desired requirements of some applications, robust xylanases (resistant to metal ions and chemicals, displaying pH, and thermostability) with specific biochemical properties for pH and temperature optima as well as, high specific activity are required, which would require purification of appropriate candidate enzymes^28^. Purification of xylanases is necessary to remove contamination by proteins and other enzymes in the culture medium, such as cellulases, as well as compounds derived from hydrolysis of the substrate. These contaminants can complicate activity assays, protein quantification, and physicochemical assays^29^. Purification and physicochemical characterization (activity and stability at various pH and temperatures) of pure xylanases provide information on the enzyme's structural and functional features, which may be used to assess its application potential^29^. Purification should be centred on attaining the highest yield while retaining the highest possible enzymatic activity and purity^30^.

Xylanases are required in large quantities for industrial-level applications because they not only have application in several processes but possess the necessary characteristics to withstand harsh conditions during these industrial processes. As a result, there is a need to select microorganisms that produce high levels of xylanases with appropriate properties, followed by optimization of growth which would lead to higher levels of enzymes^19^. There are several reports available on the optimization of media components and the physical growth parameters for the production and purification of xylanases for various applications using different substrates^8,12,21^. Xylanase production by *Trichoderma reesei* SAF3^31^ and *Trichoderma stromaticum* AM7^32^ was increased by optimization. While there are reports on multiple other species, there were very few reports on thermostable *Trichoderma harzianum* xylanases in literature.

Therefore, in the present study, a recently isolated and characterized fungal strain, *T. harzianum*, producing a thermophilic and acidic xylanase was the subject of study. The major focus of this study was to employ statistical design strategies to optimize xylanase production. Although this is not a novel approach, its application to a novel thermophilic and acidic xylanase is. We also report on the purification and characterization of the *T. harzianum* xylanase, to determine its applicability for future studies in animal feed improvement.

## Materials and methods

### Microbial strain

The *T. harzianum* strain was selected from a previous screening study as the candidate for xylanase production^33^. Fungal cultures were streaked on the PDA plates and slants, grown for 5 days at 30 °C followed by the addition of sterile mineral oil to cover the fungal mycelium and storage at 4 °C. Long-term stocks were prepared by washing fungal spores from the 5-day PDA plates with distilled water and adding 50% glycerol in a 1:1 ratio to the spore suspension and storing at − 20 °C and − 80 °C.

### Medium and cultivation

Nutrient salt solution (NSS) used for xylanase production comprised [(g/L): (0.005 g) CaCl, (0.23 g) KH_2_PO_4_, (0.05 g) MgSO_4_, (0.005 g) NaNO_3_, (0.002 g) ZnSO_4_, (0.009 g) FeSO_4_, (0.23 g) KCl, (7 g) peptone, and (20 g) wheat bran]. Erlenmeyer flasks (250 ml) containing 50 ml of the medium were each inoculated with two 5 mm fungal plugs from a 5-day-old plate culture and incubated at 30 °C at 200 rpm for 7 days in a shaker (New Brunswick Innova 44, Germany). Cultured media were removed after the incubation period and the cell-free supernatant was recovered by centrifuging samples at 16,873 × *g* for 10 min (Eppendorf centrifuge 5418, Germany). Xylanase activity was determined as described below in the xylanase assay method (“Xylanase assay”).

### Xylanase assay

Xylanase activity was quantified using the 3,5-dintrosalicylic acid (DNS) assay for reducing sugars according to the method by Miller^34^. The reaction included 600 µl of 1% (w/v) of beechwood xylan (1 g in 100 ml of 50 mM citrate buffer pH 5.0) in 15 ml test tubes to which 66.67 µl of the culture supernatant was added. The reaction mixture was incubated in a water bath at 55 °C for 15 min and terminated by the addition of 1 ml DNS reagent followed by heating for 5 min at 100 °C in a water bath. One unit (U) of xylanase was defined as the amount of enzyme that released 1 µmol xylose as reducing sugar equivalents per min under the specified assay conditions.

### Optimization of xylanase production: one factor at a time (OFAT)

To optimize the growth parameters, OFAT was used to evaluate the effect of a single parameter at a time performed in earlier study^33^. The enzyme activity was obtained to determine the optimal yield and was reported in previous studies^33^.

### Statistical optimization, experimental design, and data analysis

#### Plackett–Burman design (PBD)

Six variables were selected for this study: Incubation temperature (X_1_), Incubation time (X_2_), pH (X_3_), Agitation (X_4_), Wheat bran (X_5_), and Ammonium sulphate (X_6_) (Table 1). The total number of experimental runs carried out for the six variables was twelve^35^. Each variable was represented by a high level denoted by ‘+’ and a low level denoted by ‘−’. The high level of each variable was sufficiently far from the low level so that any significant effect would be observed. The experimental runs were performed in duplicate and an average of the results was reported Table 2 represents the PBD based on the first-order polynomial model Eq. (1):

1 $${\text{Y}} = \beta_{0} \sum \beta_{i} {\rm X}_{{\text{i}}} ,$$

where Y is defined as the response (peak area and retention factor), *β*_0_ is the model intercept, *β*_i_ is the linear coefficient and Χ_i_ is the level of the independent variable. The PBD was analyzed using R studio software^36^ to estimate the significant factors. ANOVA was performed to determine the *p*-values as well as the R coefficients to check the significance and fit of the regression model. Screened parameters were represented on a Pareto chart of standardized effects. The effect of each variable was analyzed and the ones with the highest influence on the production of xylanase were selected for the second level optimization by BBD of RSM.

**Table 1:** Experimental variables and levels used in the PBD for optimal xylanase production by the *Trichoderma harzianum* strain.

Variables	Symbol code	Units	Experimental values	
			Low level (− 1)	High level (+ 1)
Incubation temperature	X_1_	°C	40	80
Incubation time	X_2_	Days (d)	2	6
pH	X_3_	–	4	8
Agitation	X_4_	rpm	140	180
Wheat bran (carbon source)	X_5_	%	0.8	1.2
Ammonium sulphate (nitrogen source)	X_6_	%	1.0	1.4

**Table 2:** Plackett–Burman design matrix for screening of six medium components for xylanase production by the *Trichoderma harzianum* strain.

Run no	Variable level						Enzyme activity (U/ml)	
	X_1_	X_2_	X_3_	X_4_	X_5_	X_6_	Observed	Predicted
1	80 (+)	6 (+)	4 (−)	180 (+)	1.2(+)	1.4 (+)	27.1	32.3
2	40 (−)	6 (+)	8 (+)	140 (−)	1.2 (+)	1.4 (+)	68.7	68.4
3	80 (+)	2 (−)	8 (+)	180 (+)	0.8 (−)	1.4 (+)	19.3	17.5
4	40 (−)	6 (+)	4 (−)	180 (+)	1.2 (+)	1.0 (−)	29.3	27.8
5	40 (−)	2 (−)	8 (+)	140 (−)	1.2 (+)	1.4 (+)	32.5	32.6
6	40 (−)	2 (−)	4 (−)	180 (+)	0.8 (−)	1.4 (+)	8.8	9.9
7	80 (+)	2 (−)	4 (−)	140 (−)	1.2 (+)	1.0 (−)	25.6	20.5
8	80 (+)	6 (+)	4 (−)	140 (−)	0.8 (−)	1.4 (+)	23.1	18.2
9	80 (+)	6 (+)	8 (+)	140 (−)	0.8 (−)	1.0 (−)	16.2	21.2
10	40 (−)	6 (+)	8 (+)	180 (+)	0.8 (−)	1.0 (−)	34.8	30.0
11	80 (+)	2 (−)	8 (+)	180 (+)	1.2 (+)	1.0 (−)	16.7	18.3
12	40 (−)	2 (−)	4 (−)	140 (−)	0.8 (−)	1.0 (−)	9.8	15.2

X_1_: Incubation temperature.

X2: Incubation time.

X_3_: pH.

X_4_: Agitation.

X_5_: Wheat bran.

X_6_: Ammonium sulphate.

#### Optimization of the significant variables using response surface methodology (RSM)

The BBD was used to elucidate the main interaction and quadratic effects of the three significant variables arising from the PBD, with replicated centre points^21^. The experimental design and statistical analysis were performed using R Studio^36^. A three-level three-factor BBD was used to evaluate the combined effect of the three significant independent variables, Incubation time (X_2_), pH (X_3_), and wheat bran (X_5_) (Table 3). The design consisted of 16 combinations, including three replicates of the centre point as shown in Table 4. After the experimental runs were completed, the average xylanase activities were taken as the response (Y). A multiple regression analysis of the data was carried out to obtain an empirical model that relates the response measured to the independent variables^37^. The second-order polynomial Eq. (2) is shown below:

2 $${\text{Y}} = \beta_{0} + \sum \beta_{{1}} {\rm X}_{{1}} + \sum \beta_{{2}} {\rm X}_{{2}} + \sum \beta_{{{12}}} {\rm X}_{{1}} {\rm X}_{{2}} ,$$

where Y represents the response variable (peak area), *β*_0_ is the interception coefficient, *β*_1_ is the coefficient for the linear effects, *β*_2_ is the coefficient for the quadratic effect, *β*_12_ are interaction coefficient and Χ_1_ Χ_2_ is the coded independent variables that influence the response variable Y. The response in each run was the average of duplicates. In this experimental design, data were analyzed by one-way ANOVA with Tukey’s multiple comparison test (p ≤ 0.05) using R studio^36^, and ggplot2 was used for the generation of 3D response surface and contour plots^38^.

**Table 3:** Experimental codes and levels of independent variables in the RSM for optimal xylanase production by the *Trichoderma harzianum* strain.

Variables	Symbol code	Experimental values		
		Low (−1)	Zero (0)	High (+ 1)
Incubation time (d)	X_2_	4	5	6
pH	X_3_	4	5	6
Carbon source Wheat bran (%)	X_5_	0.8	1	1.2

**Table 4:** Experimental design for the BBD model for three significant independent variables (Incubation time, pH, and wheat bran) tested and predicted and observed responses for xylanase production.

**Run order**	Experimental value (coded value)			Enzyme activity (U/ml)	
	Incubation time (d)	pH	Wheat bran %	Observed	Predicted
1	4 (−)	4 (−)	1 (0)	97.09	86.35
2	6 (+)	4 (−)	1 (0)	89.76	87.11
3	4 (−)	6 (+)	1 (0)	98.65	107.01
4	6 (+)	6 (+)	1 (0)	109.22	102.35
5	4 (−)	5 (0)	0.8 (−)	90.82	97.68
6	6 (+)	5 (0)	0.8 (−)	101.32	108.72
7	4 (−)	5 (0)	1.2 (+)	103.37	88.34
8	6 (+)	5 (0)	1.2 (+)	153.80	134.75
9	5 (0)	4 (−)	0.8 (−)	60.01	58.78
10	5 (0)	6 (+)	0.8 (−)	66.92	74.32
11	5 (0)	4 (−)	1.2 (+)	82.99	69.65
12	5 (0)	6 (+)	1.2 (+)	116.74	64.98
13	5 (0)	5 (0)	1 (0)	29.68	37.85
14	5 (0)	5 (0)	1 (0)	33.23	37.85
15	5 (0)	5 (0)	1 (0)	27.38	37.85
16	5 (0)	5 (0)	1 (0)	42.30	37.85

### Scaled-up production in the optimized medium

The optimized parameters for each factor from the statistical design experiments were implemented for the scaled-up production of the xylanases. The nutrient salt solution was prepared as previously described (“Medium and cultivation”) and supplemented with the optimized wheat bran and ammonium sulphate concentrations. Erlenmeyer flasks (2 l) containing 400 ml of the medium were each inoculated with two 5 mm fungal plugs from a 5-day-old plate culture and incubated at the optimized parameters in a shaker (New Brunswick Innova 44, Germany). Cultured media were removed after the incubation period and the cell-free supernatant was recovered by centrifuging samples at 16,873 × *g* for 10 min (Eppendorf centrifuge 5418, Germany). Xylanase activity was determined as described in “Xylanase assay”.

### Purification of xylanase

All purification steps were carried out at 4 °C. Partial purification of the xylanase was carried out by ammonium sulphate precipitation (20–80%). The pellets were dissolved in 50 mM citrate buffer pH 5.0 and subjected to dialysis overnight in the same buffer. The fraction that resulted in the highest activity, was concentrated in 3 kDa Amicon centrifugal tubes, the protein precipitate dissolved in 50 mM citrate buffer pH 5.0 buffer and loaded onto an anion exchange column (HiTrap Q FF 5 ml) which was connected to the AKTA Purifier (AKTA Purifier, GE Healthcare Bio-Science, AB75184, Uppsala Sweden). Before loading the sample, the column was equilibrated with 20 mM Tris buffer, (pH 8.0). The enzyme was eluted using a 0–2 M sodium chloride gradient at a flow rate of 1.5 ml/min. Fractions were collected and those displaying xylanase activity were pooled, concentrated, and dialyzed against a 50 mM citrate buffer (pH 5.0), to be used for further characterization of the enzyme. Protein concentration was measured by the Bradford method^39^ using bovine serum albumin as the standard. The samples were separated on a 12% SDS-polyacrylamide gel according to Laemmli^40^. Native PAGE was performed with 1% xylan as the substrate. Once electrophoresis was completed, the gel was incubated in 50 mM citrate buffer pH 5.0 at the optimum temperature (70 °C) for 20 min and thereafter stained with 0.1% Congo red solution for 30 min and destained in 1 M NaCl until clearance bands representing xylanase activity were obtained.

### Characterization of purified xylanase

#### Effect of pH and temperature on xylanase activity

The pH optimum was determined by measuring enzyme activity between pH 4.0 and 10.0 The following buffers were used: 0.1 M sodium citrate buffer (pH 3.0–5.0), 0.1 M potassium phosphate buffer (pH 6.0–8.0) and 0.1 M Glycine NaOH buffer (pH 9.0–10.0)^41^. Enzyme assays were conducted as previously described (“Xylanase assay”). For determination of the optimum temperature, the reactions were carried out at the optimum pH between 40 to 80 °C with intervals of 5 °C.

#### pH and thermostability

The pH stability of the enzyme was determined by incubating the enzyme in the optimal pH buffer for 4 h at 55 °C with aliquots removed every 30 min. A substrate control was also incubated for 4 h. Thereafter, xylanase activity was assayed using the DNS method and reported as residual activity (%). Temperature stability was determined by incubating the enzyme in the optimal pH buffer at optimal temperature for 4 h with aliquots collected every 30 min. The activity was assayed and reported as residual activity (%).

#### Effect of metallic ions and different solvents on xylanase activity

The effect of metallic ions (CaCl_2_, CoCl_2_, FeSO_4_, MgSO_4_, MnSO_4_, and ZnSO_4_) and chemical agents (SDS, DMSO, and EDTA) on enzyme activity was evaluated at two concentrations: 2 mM and 10 mM. The residual activity was measured using the standard assay conditions. The activity in the absence of metallic ions or inhibitors was taken as the control (100%)^42^.

#### Substrate specificity

The specificity of the purified xylanase was verified by assaying the activity using various substrates, viz., beechwood xylan, birchwood xylan, xylan from Larchwood, wheat arabinoxylan (soluble and insoluble), carboxymethylcellulose (CMC) and Avicel. Substrates (1% w/v) were suspended in 50 mM citrate buffer (pH 6.0) and incubated with the purified enzyme at 65 °C for 15 min and thereafter the xylanase activity was determined by the DNS method as described previously (“Xylanase assay”)^43^.

#### Kinetic parameters

The *K*_*m*_ and *V*_*max*_ values for the xylanase were determined by measuring the enzymatic activity using different concentrations of the xylan substrate (1–20 mg/ml). The activity was measured under standard assay conditions as described previously. The Michaelis-Menton and Lineweaver- Burk plots were acquired to determine *K*_*m*_ and *V*_*max*_.

### Equipment and settings

Neither image acquisition tools nor image processing software packages were used for the figures in this study. For Fig. 7, processing such as changing brightness and contrast was applied equally across the entire image and applied equally to the controls. The contrast does not allow for any data to disappear. There were no excessive manipulations, such as processing to emphasize one region in the image at the expense of others.

## Results and discussion

### Screening of significant medium constituents for xylanase production

**Figure 1:**
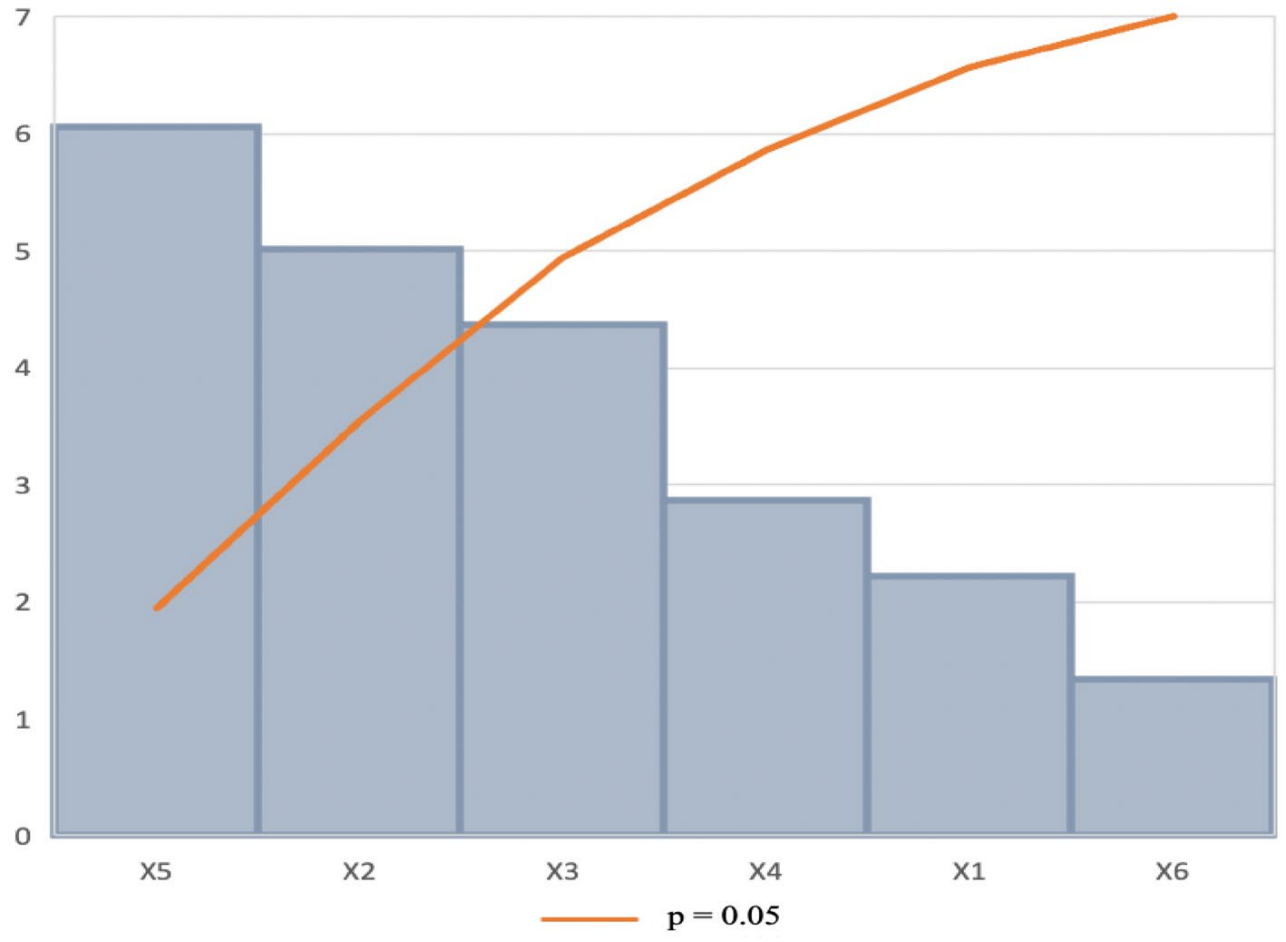
Pareto chart of standardized effects for the production of xylanase. Incubation temperature (X_1_), incubation time (X_2_), pH (X_3_), agitation (X_4_), wheat bran (X_5_), ammonium sulphate (X_6_).

The rows in Table 2 represent the twelve different experiments. The data obtained from the PBD runs indicate a wide variation in xylanase activity from 9.8 to 68.7 U/ml across the twelve runs. This variation demonstrated that the effect of the medium and culture conditions on the production of xylanase was significant (p < 0.05). The *R*^2^, or coefficient of determination, is the percentage of response variance that can be ascribed to the model rather than a random error^44^. According to Xie et al.^45^, *R*^*2*^ should be at least 90% for a model to fit well. The determination coefficient (*R*^2^) indicates that the independent variables were responsible for 97 percent of the sample variance in xylanase output, and just roughly 3% of the overall variation was not explained by the model. The greater the correlation between experimental and anticipated values, the closer R (correlation coefficient) is to 1. The value of R (0.97) indicated that the experimental data and the theoretical values predicted by the model equation were in close agreement. As indicated in Table 5, the *p*-value was used to verify the significance of each of the coefficients. The incubation period (X_2_), pH (X_3_), and wheat bran (X_5_) were all shown to have a significant (*p* < 0.05) effect on xylanase activity. The Pareto chart of standardization (Fig. 1) confirmed that these three factors significantly influenced xylanase production (*p* < 0.05), as they crossed the *p*-line. However, the other independent factors (*p* > 0.05) were generally considered insignificant.

**Table 5:** Analysis of variance (ANOVA) for six variables by PBD experiment.

	df	Sum of squares	Mean square	F-value	*p*-value
Model	6	2521.08	420.18	12.59	0.00700*
Incubation temperature (X_1_)	1	119.1	119.1	3.992	0.10220
Incubation time (X_2_)	1	610.6	610.6	20.473	0.00626*
pH (X_3_)	1	280.1	280.1	9.392	0.02796*
Agitation (X_4_)	1	166.0	166.0	5.566	0.06481
Wheat bran (X_5_)	1	1277.7	1277.7	42.839	0.00125*
Ammonium sulphate (X_6_)	1	66.3	66.3	2.222	0.19622
Residuals	5	166.92	33.39		

*df* degree of freedom.

*Significant *p*-value at p ≤ 0.05.

Adjusted R^2^ (mean coefficient of determination) = 0.97.

There is a 97% chance that the model explains the measured variations in response. The magnitude and direction of the factor coefficient in the equation clarify the influence of the six variables for xylanase production. The higher magnitude indicated a large effect on the response. The corresponding response of xylanase activity was expressed in terms of the following regression Eq. (3) derived from the Unstandardized Beta values (Table 6):

3 $${\text{Y}} = {\text{X}}_{{1}} + {\text{X}}_{{2}} + {\text{X}}_{{3}} + {\text{X}}_{{4}} + {\text{X}}_{{5}} + {\text{X}}_{{6}} ,$$

$${\text{Y }} = { 2}0.{58 } - \, 0.{\text{47X}}_{{1}} + { 4}.{\text{28X}}_{{2}} + { 1}.{\text{51X}}_{{3}} {-} \, 0.{\text{16X}}_{{4}} + { 23}.{\text{99X}}_{{5}} + { 4}.{\text{56X}}_{{6}} ,$$

where Y is defined as the peak area, X_1_ refers to the incubation temperature, X_2_ is the incubation time, X_3_ is the pH, X_4_ is the agitation, X_5_ is the wheat bran and X_6_ is the ammonium sulphate.

**Table 6:** Effect estimates for xylanase production from the results of PBD.

	Unstandardized beta	Coefficients Std.Error	Standardized coefficients beta	t-value
Model	20.58	14.45		1.42
Incubation temperature (X_1_)	− 0.47	0.11	− 0.61	− 2.22
Incubation time (X_2_)	4.28	0.85	0.57	5.02
pH (X_3_)	1.51	0.68	0.25	4.37
Agitation (X_4_)	− 0.16	0.06	− 0.33	− 2.87
Wheat bran (X_5_)	23.99	3.96	0.80	6.06
Ammonium sulphate (X_6_)	4.56	3.41	0.15	1.34

### Optimization of significant variables for xylanase production

#### Box Behnken design

**Figure 2:**
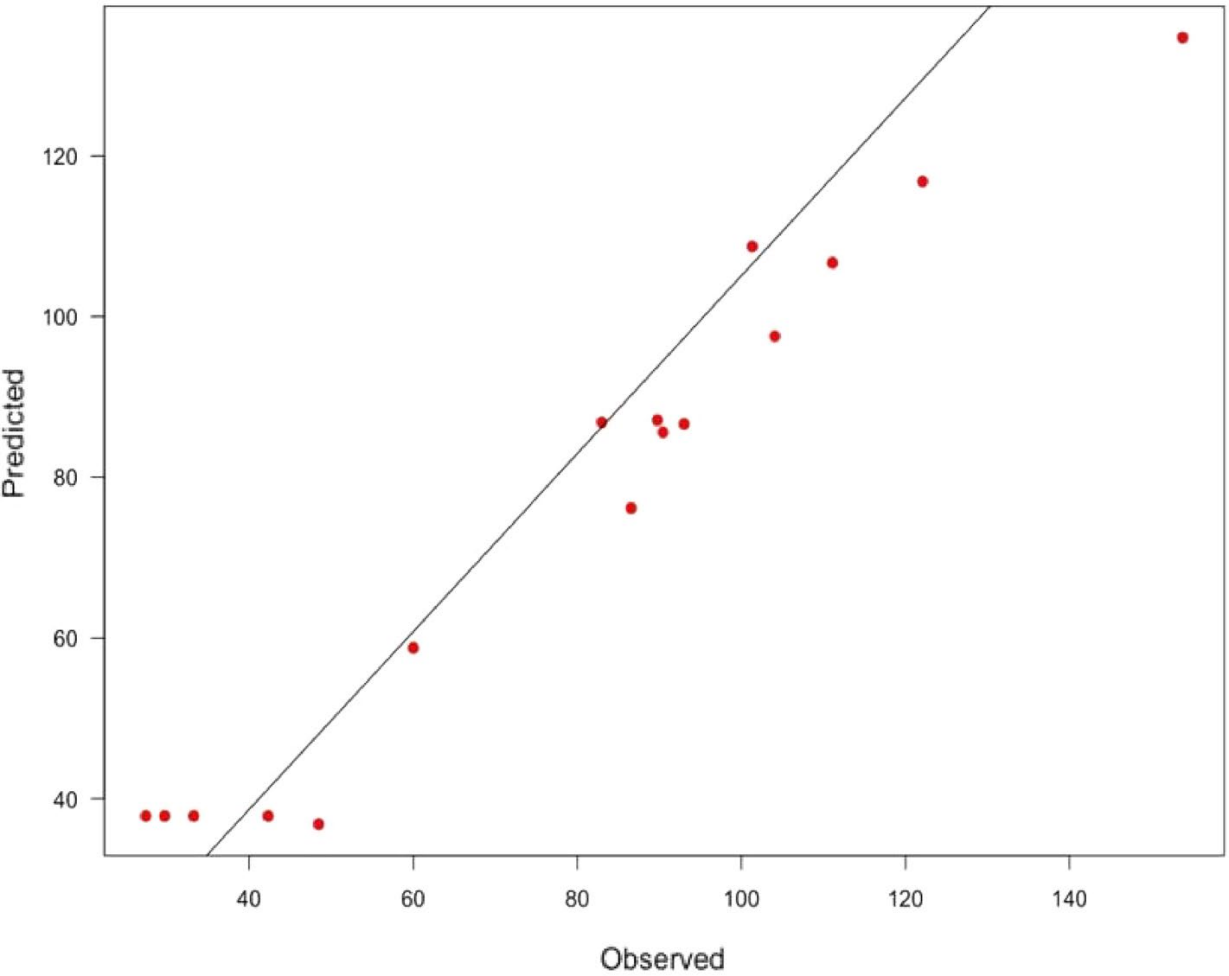
Graphical representation of the minimal difference between the actual (straight line) and predicted responses (circles) for the Response Surface Methodology Design for optimal xylanase activity.

A total of 16 runs were performed to determine the conditions for optimal xylanase production by *T. harzianu*m. A matrix was run with the three significant variables that emerged from the PBD experiments. The results for the BBD runs (Table 4) show that the lowest activity of 27.38 U/ml was obtained under zero-level conditions (5 days, pH 5.0, and 1% wheat bran) in run 15 while run 8 resulted in the highest xylanase activity of 153.80 U/ml under the following conditions: 6 days of incubation, pH 5.0, and 1.2% wheat bran. This was significantly and markedly (over four-fold)_higher (p ≥ 0.05) than the highest enzyme activities obtained during OFAT (38.50 U/ml). Long et al.^21^ confirmed a similar but lower influence of optimized parameters on xylanase production (174.46–266.70 U/ml) by *Trichoderma orientalis*. Using the quadratic equation, the predicted values were determined (Table 4). The *R*^*2*^ or coefficient of determination (0.9647, close to 1) confirmed the validity of the model, i.e., that 96.47% of the variability of the response can be expressed by the model. The value of the coefficient of adjusted determination, adjusted *R*^2^, was 0.9112 confirming that the actual values were close to the predicted values^46,47^. The correlation was confirmed by plotting the actual value curve as a function of the predicted values (Fig. 2) which shows the points distributed around the regression line. Figure 2 shows that the actual response values agreed well with the predicted response values, thus the predicted xylanase production is within the limits of the experimental factors. Therefore, the model is considered of sufficient quality^46^ with a 96.47% chance that it explains the measured variations in response.

Maximum xylanase production (153.80 U/ml) by the *T. harzianum* strain occurred in BBD run 8 under acidic conditions (pH 5.0), the higher wheat bran (1.2%), and a 5-day incubation period. Lightly lower activity can be observed for run 12 (116.74 U/ml) where the incubation period was 5 days, the wheat bran was 1.2% and the pH was 6.0. Even lower but similar enzyme activities were obtained for runs 6 (101.32 U/ml) and 7 (103.37 U/ml) where either the incubation time (4 or 6 days) or wheat bran (0.8 or 1.2%) was at their low or high levels, respectively compared to run 8 where both these parameters were at their high levels (6 days and 1.2%). This may be due to the presence of two isoforms that are maximally produced under acidic conditions. The presence of isoforms requires different periods of incubation for maximal xylanase activity and various wheat bran concentrations. In the presence of xylan, most microorganisms can produce multiple types of xylanases. Fungi are well-known for producing a wide range of xylanases (up to 30 multiple forms)^5,48^. Zhang et al.^49^ reported that three xylanase isoforms were produced by *Aspergillus fumigatus*. Multiple forms of xylanases differ in stability, catalytic efficiency, absorption, and activity on substrates^50^. Okafor et al.^51^ isolated a strain of *Penicillium chrysogenum* PCL501 from wood wastes and found that after 4 days of fermentation, wheat bran produced the highest xylanase activity of 6.47 U/ml. Abdel-Sater et al.^52^ obtained maximum xylanase production from *T. harzianum* after 8 days of fermentation whereas, Thomas et al.^53^ achieved maximum xylanase production in 4 days of fermentation by an *Aspergillus* sp.

The production of multiple forms of xylanases can be influenced by many factors, including the presence of various alleles of the same gene, variable mRNA processing, proteolytic digestion post secretion, and post-translational modifications such as glycosylation and autoaggregation^54^. Because xylanases have varying catalytic efficiencies, the production of several xylanases is particularly beneficial for the complete hydrolysis of hemicellulosic substances^55^. Generally, xylanase production is directly proportional to the duration of the fermentation time up to a certain level and then decreases, thus, incubation time affects xylanase production by fungi^56^.

#### Second-order regression and prediction

The second-order regression equation provides the xylanase activity produced by the *T. harzianum* strain as a function of incubation time (X_2_), pH (X_3_), and wheat bran (X_5_) which can be presented in the following Eq. (4):

4 $${\text{Y}} = { 44}.{91} - 0.00{\text{4X}}_{{2}} + 0.0{\text{12X}}_{{3}} + {42}.0{\text{9X}}_{{5}} - 0.00{\text{4X}}_{{{22}}} + 0.0{\text{12X}}_{{{32}}} + {42}.0{\text{9X}}_{{{52}}} + {\text{X}}_{{2}} {\text{X}}_{{3}} + {\text{X}}_{{2}} {\text{X}}_{{5}} + {\text{ X}}_{{3}} {\text{X}}_{{5}} ,$$

where Y is the peak area, X_2_ is the incubation time, X_3_ is the pH and X_5_ is the wheat bran concentration. The statistically insignificant parameters (*p* > 0.05) and their interactions were omitted from the equation. The model constants and coefficients were generated using the unstandardized beta values.

#### ANOVA and Pareto chart

**Figure 3:**
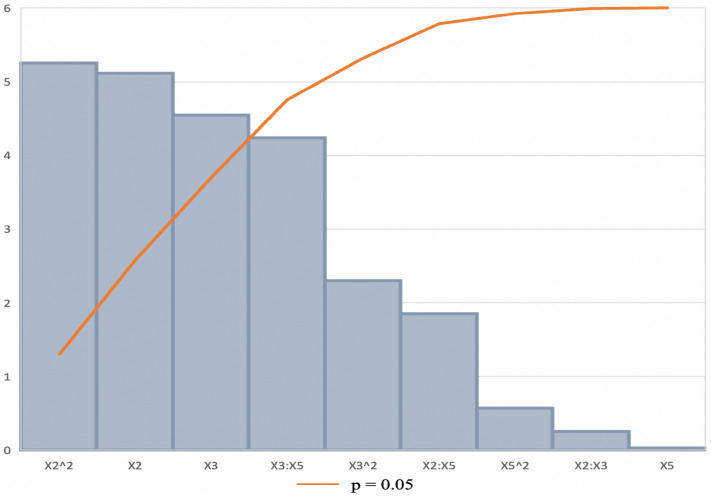
Pareto chart standardized effects of nine interactive factors affecting the production of xylanase optimization. Incubation temperature (X_1_), incubation time (X_2_), pH (X_3_), agitation (X_4_), wheat bran (X_5_), ammonium sulphate (X_6_).

The “Lack of fit *p*-value” (Table 7 ) was insignificant as the *p*-value was greater than 0.05, however, literature shows this *p*-value (> 0.05) is considered acceptable^57^. According to Bezerra et al.^58^, significant regression and a non-significant lack of fit present in the model were well-fitted to the experiments. Based on this, the regression equation can be validated^59^. ANOVA was performed to determine the *p*-values (Table 7). This showed the model, the linear and square terms for X_2_ (Incubation time), and the interaction between X_3_ (pH) and X_5_ (Wheat bran) as well as the linear terms of X_3_ (pH) to be significant as the *p*-values were 0.00001, 0.0001, 0.00005, 0.02042 and 0.01137, respectively. The Pareto chart of standardization histogram graph (Fig. 3) also showed that Incubation time (X_2_, X_2_^2^), the interaction between pH and wheat bran (X_3,_ X_5_), and pH (X_3_) was significant (*p* < 0.05), as it crosses the *p*-line.

**Table 7:** Analysis of variance (ANOVA): and regression coefficients of the response surface quadratic model for the response variables for xylanase production by *Trichoderma harzianum* strain.

	Estimate	Std. Error	t value	*p*-value
Model	2736.80	227.41	12.03	0.00001*
Incubation time (X_2_)	− 555.80	43.97	− 12.64	0.00001*
pH (X_3_)	− 178.57	49.62	− 3.60	0.01137*
Wheat bran (X_5_)	− 1897.16	219.83	− 8.63	0.00013
Incubation time (X_2_): pH (X_3_)	4.48	3.78	1.18	0.28169
Incubation time (X_2_): Wheat bran (X_5_)	12.08	25.78	0.47	0.65598
pH (X_3_): Wheat bran (X_5_)	33.55	18.92	1.77	0.02042*
Incubation time (X_2_)^2^	52.50	4.17	12.59	0.00005*
pH (X_3_)^2^	13.04	4.17	3.13	0.12655
Wheat bran (X_5_)^2^	887.04	104.24	8.51	0.00014

*Significant *p*-value at *p* ≤ 0.05.

Adjusted *R*^2^ = 0.9117.

Lack of fit *p*-value = 0.3741.

#### Interaction of variables

**Figure 4:**
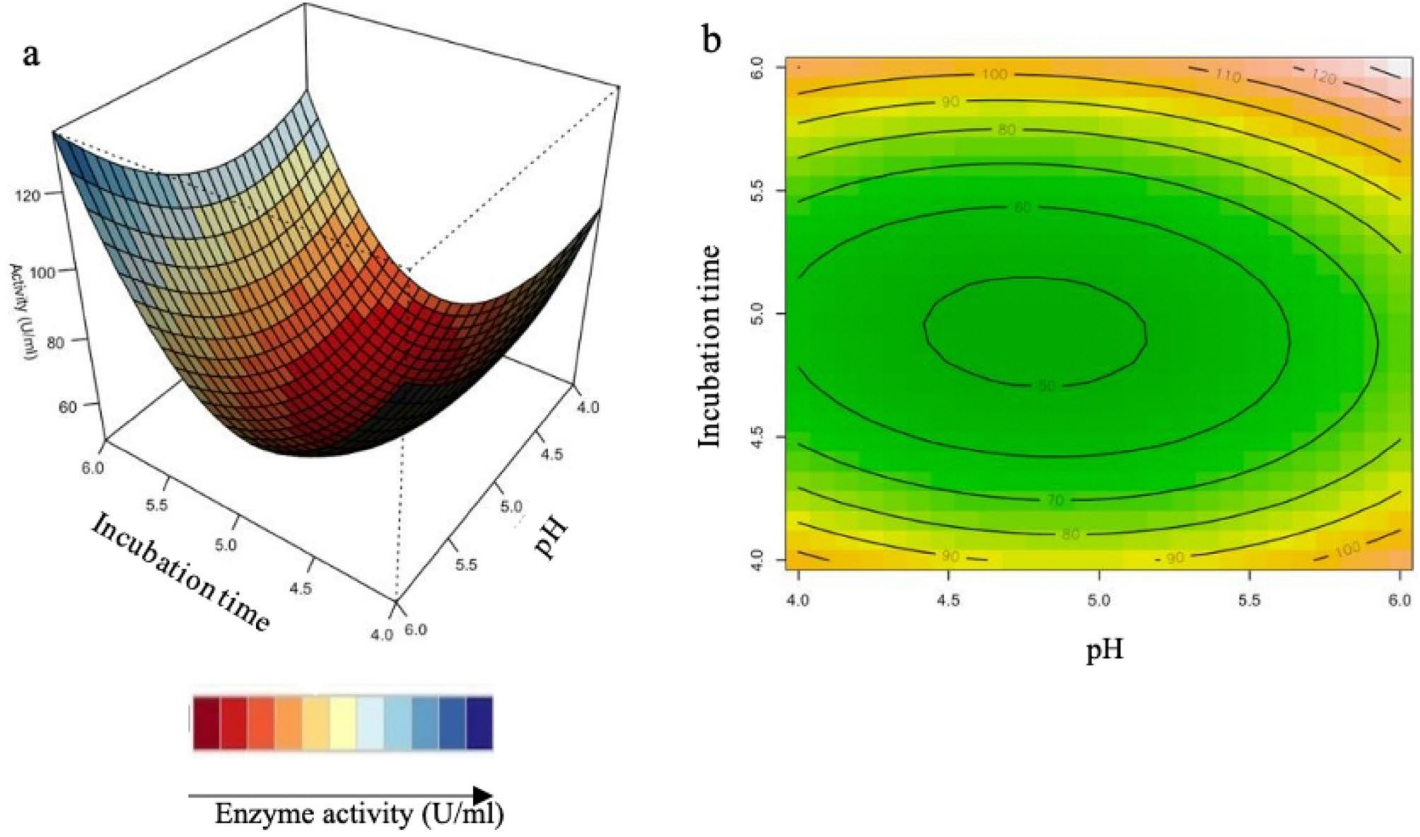
3D-response surface plots (**a**) and contour plots (**b**) of the combined effects of Incubation time (X_2_) and pH (X_3_) on xylanase production by *Trichoderma harzianum* strain.

**Figure 5:**
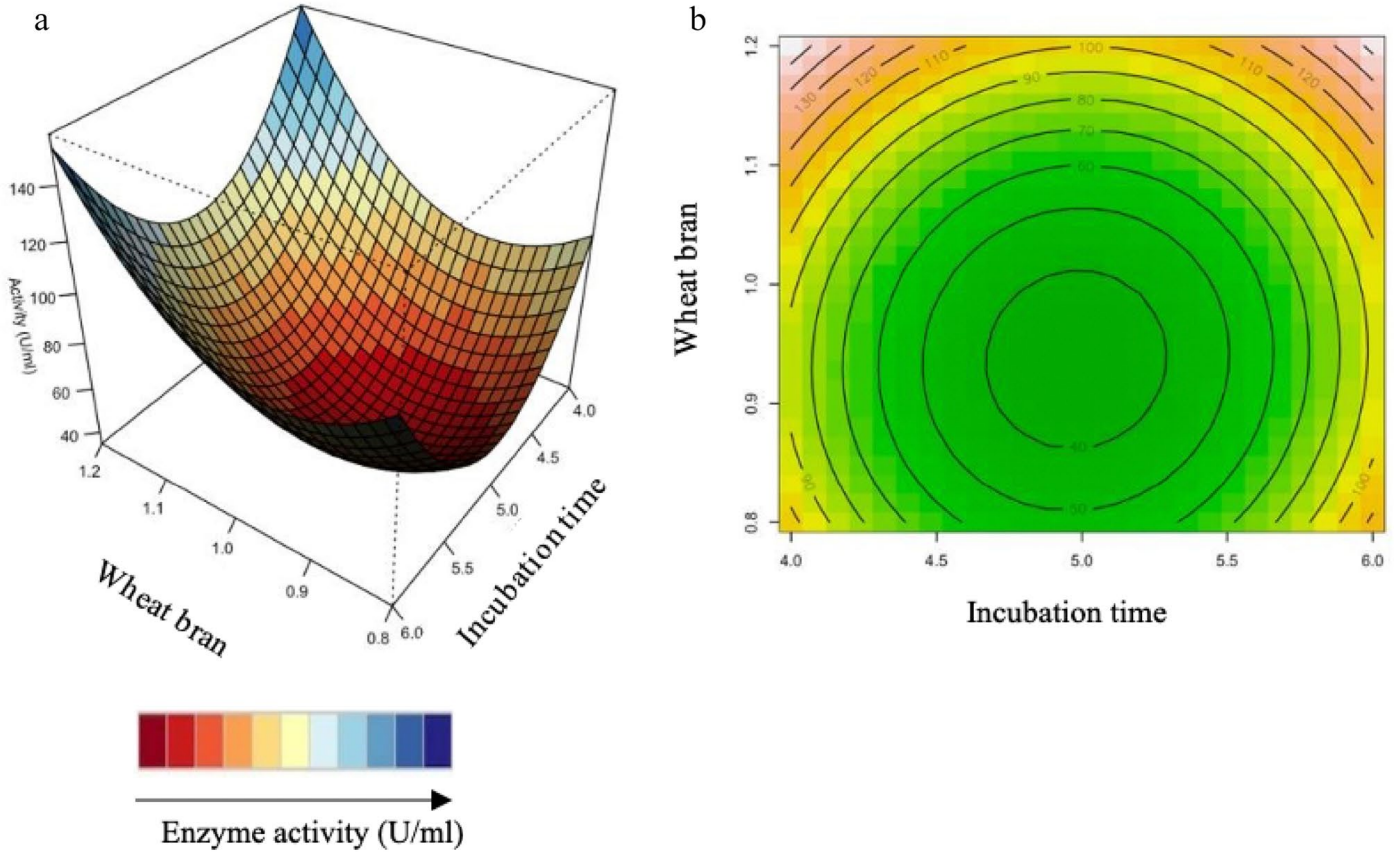
3D-response surface plots (**a**) and contour plots (**b**) of the combined effects of Incubation time (X_2_) and wheat bran (X_5_) on xylanase production by *Trichoderma harzianum* strain.

**Figure 6:**
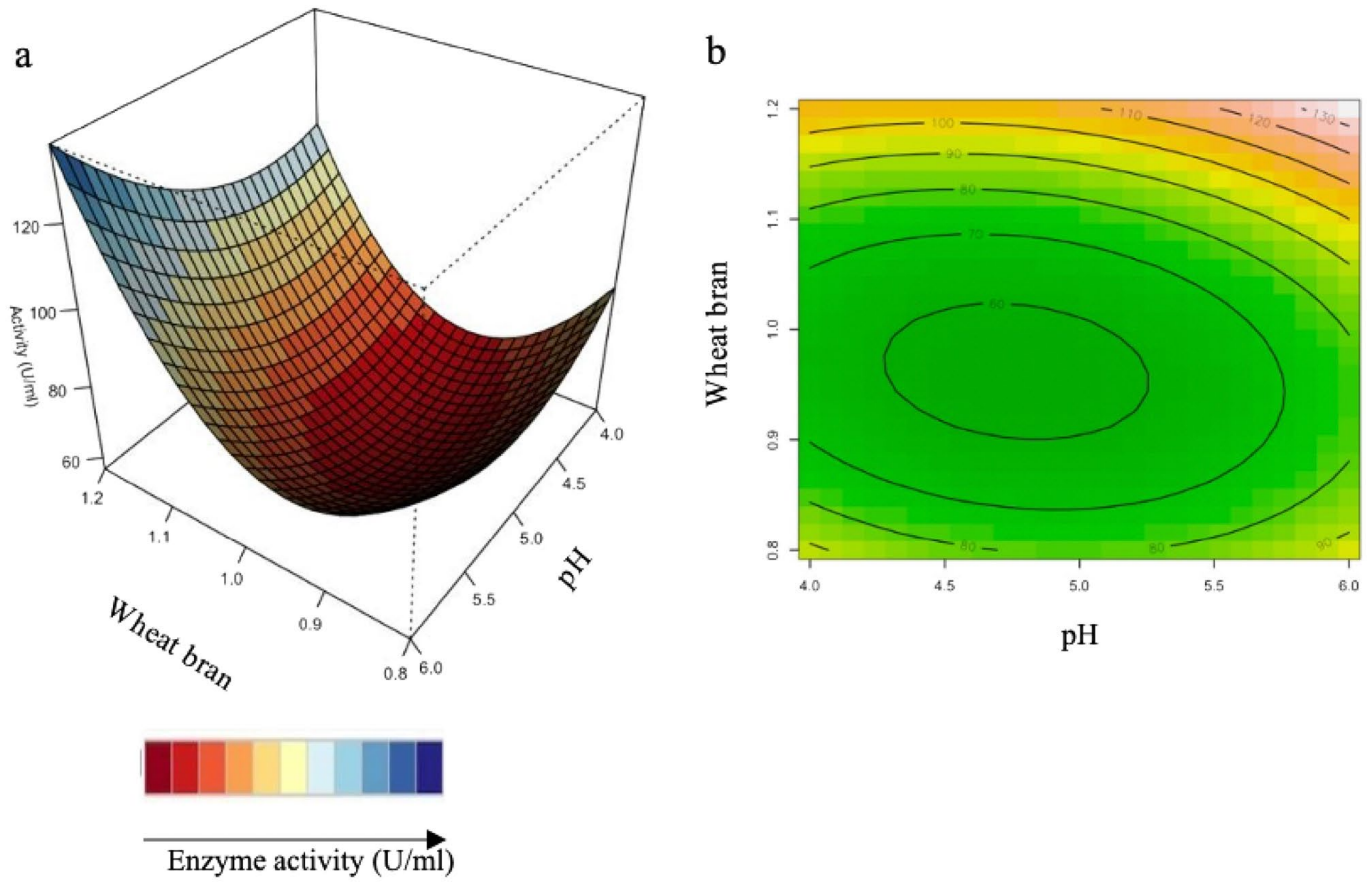
3D-response surface plots (**a**) and contour plots (**b**) of the combined effects of pH (X_3_) and wheat bran (X_5_) on xylanase production by *Trichoderma harzianum* strain.

The relationship between the parameters and the responses can be understood by studying the three-dimensional (3D) response surface plots for xylanase activity generated from the quadratic model. The 3D response surface plot can also be used to determine the optimum level of each variable for xylanase activity (Figs. 4, 5, 6). While maintaining other variables at their optimal level, the Z-axis (referring to xylanase activity) versus any two variables was constructed in the response surface plot.

Figure 4a,b illustrate the combined effects of incubation time and pH xylanase activity increases at a high pH and shorter incubation time. Figure 4b illustrates the contour plot, which shows that high enzyme activity was obtained at the shortest (4 days) and longest period (6 days) of incubation in acidic (4.0–6.0) conditions. Yadav et al.^29^, reported similar pH conditions for optimization of xylanse production from *Anoxybacillus kamchatkensis* NASTPD13.

Figure 5a,b show that xylanase production is directly proportional to incubation time and wheat bran. This could be due to higher levels of degradation of xylan present in the wheat bran by *T. harzianum*. In Fig. 5b, it is apparent that the xylanase activity is highest at high concentrations of wheat bran with the shortest (4 days) and longest period (6 days) of incubation. Previous studies showed the time course during the OFAT approach, being favourable at 4 days and 6 days of incubation with the optimum being at 5 days^33^. The RSM plots correspond with the OFAT results, as the plots show the highest xylanase activity obtained at high wheat bran between 4 and 6 days. Simultaneously, it was highlighted by Beg et al.^60^ that wheat bran could effectively induce higher xylanase production by *Aspergillus awamori.* Li et al.^61^ also reported the importance of the substrate concentrations for xylanase production by *A. awamori.* The facts mentioned here, correspond to the reports by Cui and Zhao^62^, as they mention that the enzymes, which are involved in substrate degradation, were generally mostly inducible. These were formed only when the substrate it correlates with, was present in the nutrient salt solution.

Figure 6 shows the highest xylanase activity obtained with high wheat bran and the entire pH range tested. However, at lower wheat bran concentrations, higher xylanase activity can be observed at the pH extremes tested (at approximately pH 4 and pH 6). Figure 6b illustrates, at high wheat bran concentration and over a wide pH range with the highest activity obtained at the highest pH and wheat bran concentration. The interaction between the pH and wheat bran (130 U/ml) and between incubation time and wheat bran (130 U/ml) had the highest effect on xylanase activity compared to the interaction between incubation time and pH (120 U/ml).

The study successfully demonstrated a notable increase in enzyme activity using the statistically designed experiments compared to OFAT. It was also demonstrated that multiple forms of xylanase were produced (isoforms) based on variations in the growth and media conditions. Based on Table 4, there could potentially be 5 different isoforms. High xylanase activity was observed for runs 4, 6, 7, 8, and 12. Supplementary Fig. 6 representing these RSM runs indicates the presence of isoforms by several zones of clearance on the substrate native PAGE gels.

Multiple forms of xylanases with different pH optima could be beneficial for animal feed improvement^13^. Xylanase is used to reduce the viscosity of the feed and improve the absorption of nutrients in the digestive tract of animals. The enzymes could be applied before the pelleting process, which operates between pH 4.0 and 6.0 thus requiring enzymes that are active within this pH range. Most xylanases reported to date are optimally active in the acidic or neutral pH range. Xylanases with acidic pH optima could potentially also be useful for applications containing waste, as a method of waste management, and as a feedstock for fermentable sugars^63^.

### Scaled-up fermentation in optimized conditions for further studies

The xylanase enzyme was produced on a larger scale for further studies. The enzyme was produced at pH 5.0 for 6 days of incubation and with 1.2% wheat bran. The enzyme activity was determined in order to compare the activities to the smaller scale production. The enzyme activity obtained was 152.78 U/ml which was similar to the enzyme production on a smaller scale (153.80 U/ml).

### Purification of xylanase from the *T. harzianum* isolate and zymography

**Figure 7:**
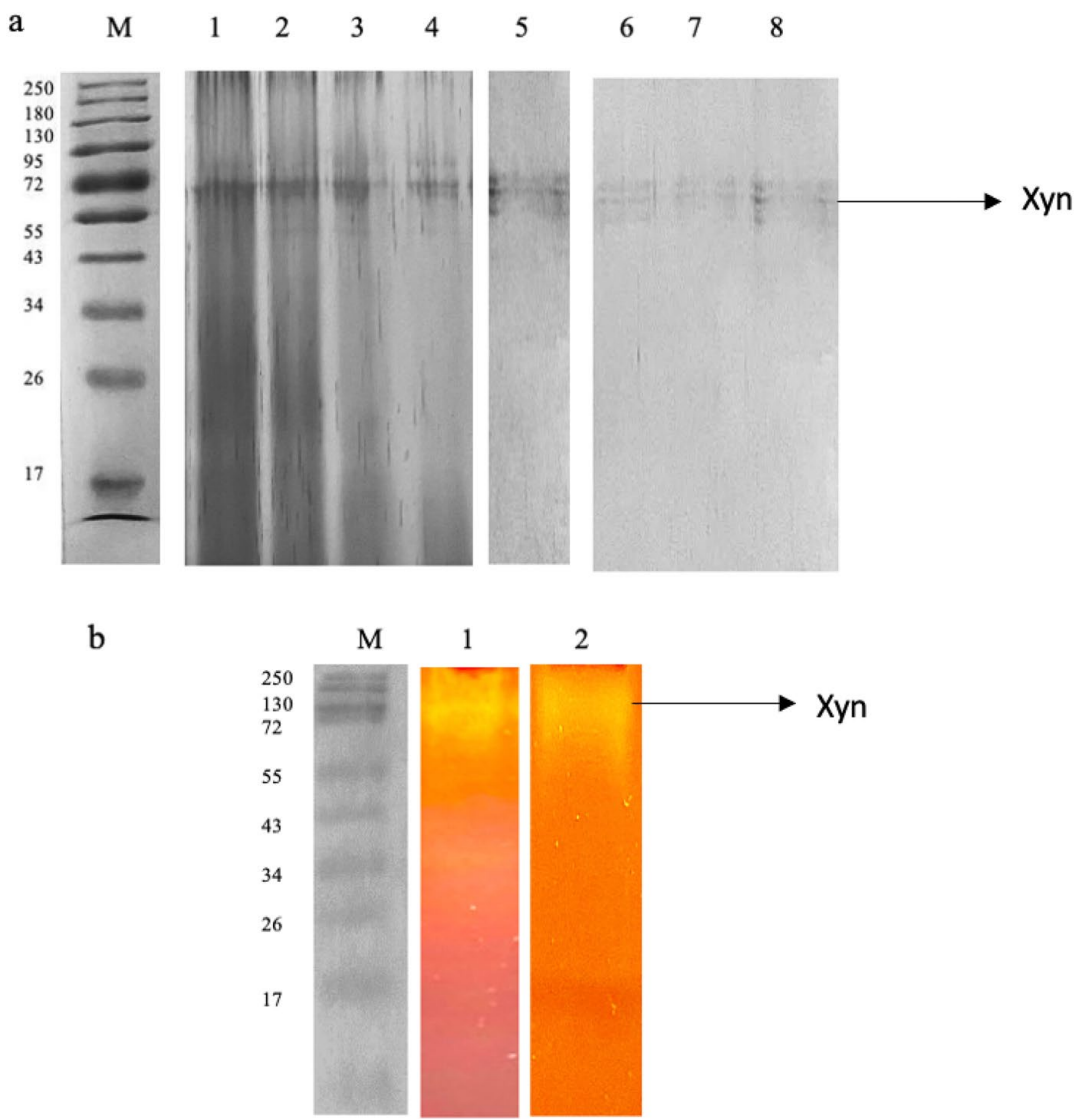
A 12% SDS PAGE (**a**) and Native substrate-PAGE (**b**) analysis of purified xylanase. 12% SDS PAGE (cropped) represents Lane M: Molecular weight marker (Thermoscientific, USA), 1–4: 50, 60, 70, and 80% ammonium sulphate fractions (Coomassie-stained), and 5–8: Purified xylanase from *Trichoderma harzianum* (Xyn). Native substrate-PAGE (cropped) represents Lane M: Molecular weight marker (Thermoscientific, USA), 1: 50% Ammonium sulphate fraction showing zone of clearance, and Lane 2: Purified xylanase (Xyn) from *Trichoderma harzianum* on native substrate gel showing zone of clearance. The original gels are presented in Supplementary Figs. 1–5.

The xylanase from *T. harzianum* was purified using ammonium sulphate precipitation, dialysis, and chromatographic methods in combination. Table 8 summarizes the purification stages. The enzyme was fractionated with the following ammonium sulphate saturations: (0–19%, 20–29%, 30–39%, 40–49%, 50–59%, 60–69% and 70–79%). The 70–79% saturation fraction resulted in significantly high xylanase activity with a recovery of 20.31% enzyme activity. The 50–59%, 60–69%, and fractions also showed relatively high recovery of enzyme activity (18.73%, and 17.48%, respectively) whereas 10.42% enzyme was recovered in the 40–49% fraction (Table 8) therefore these fractions were further studied to confirm if they were isoforms. The active fractions were then dialyzed at 4 °C overnight to remove the salts, and the enzyme was loaded onto DEAE Sephadex for further purification. A 0–2 M sodium chloride concentration gradient was used to elute the bound protein. Xylanase activity was measured in both bound and unbound protein fractions. The primary peak eluted at 0.5 M sodium chloride and the corresponding fraction had a specific activity of 254.62 mol/mg, and a 2.52-fold purity. Furthermore, a single band with a molecular weight of 72 kDa was evident on SDS-PAGE gels of the purified enzyme (50%) (Fig. 7a). The other ammonium sulphate fractions (60–79%) also have the same molecular weight protein (72 kDa). To assess the activity/purity, the purified xylanase was subjected to zymogram analysis by substrate native-PAGE (1% beechwood xylan). The xylanolytic activity of the enzyme was indicated by clear zones in the gel after Congo-red staining (Fig. 7b). Purified preparations of enzymes are a requisite for their application as well as elucidating their basic characteristics and mechanisms. Based on the high molecular weight of the purified enzyme, it can be tentatively inferred that it may belong to the GH10 family since enzymes belonging to this family feature a larger molecular weight^63^. Enzymes are also classified based on their catalytic reactions. Based on the sequence similarities of amino acids, xylanases are classified into glycosyl hydrolase (GH) families 10 (GH10) and 11 (GH11)^2^. Family GH10 contains xylanases of high molecular mass (> 30 kDa) with a (β/α)_8_ barrel structure and acidic *pI* values, while GH11 include are the low-molecular-weight endoxylanases which are divided into alkaline *pI* and acidic *pI* xylanases^2^.

**Table 8:** Purification table for xylanase from *Trichoderma harzianum* strain.

AS fraction (%)	Total protein (mg)	Total activity (U)	Specific Activity (U/mg)	Yield (%)	Fold purity
Crude extract	95	9593	101.21	100	1.0
**Ammonium sulphate fraction**					
50–59%	12.77	2394.51	187.53	24.96	1.85
60–69%	10.45	1796.44	171.91	18.73	1.70
70–79%	10.11	1676.73	165.85	17.48	1.64
80–89%	7.02	1947.93	277.48	20.31	2.74
**Anion exchange chromatography**					
50–59%	0.40	999.90	2499.75	10.42	24.70
60–69%	0.28	636.88	2274.57	6.64	22.47
70–79%	0.16	313.67	1960.44	3.27	19.37
80–89%	0.32	700.60	2189.38	7.30	21.63

### Characterization of xylanase

#### pH optimum and stability

**Figure 8:**
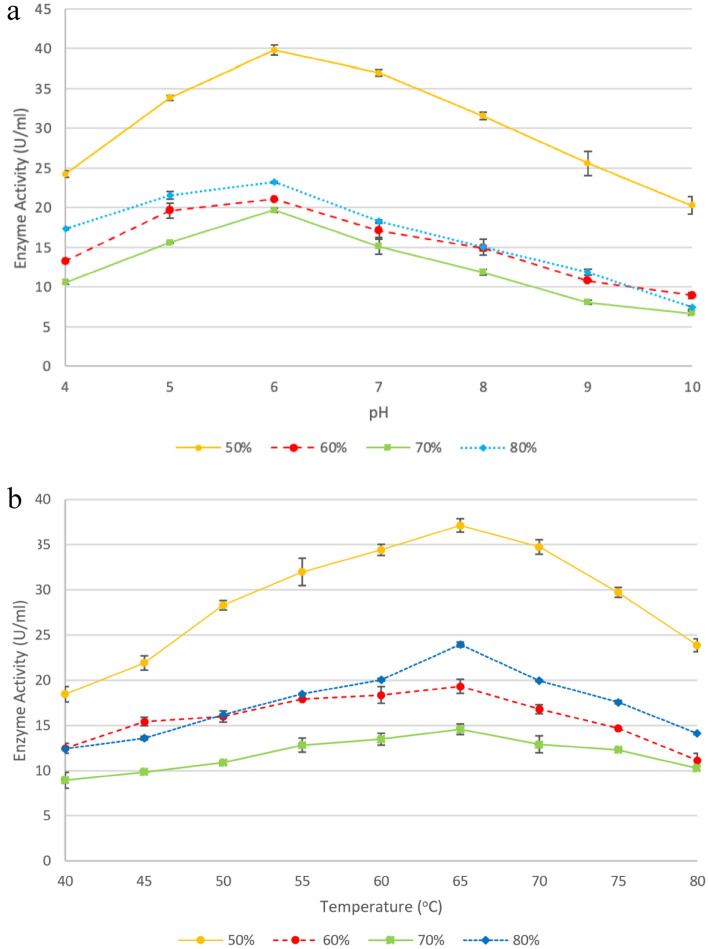
Effect of pH (**a**) and temperature (**b**) on the activity of purified xylanases (50%, 60%, 70%, and 80% ammonium sulphate fractions). Data points represent the means ± SD (n = 4).

**Figure 9:**
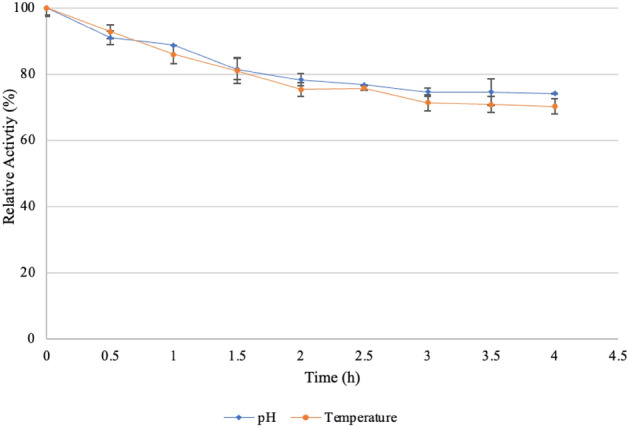
pH and temperature stability of purified xylanases (50% ammonium sulphate fraction) produced by the *Trichoderma harzianum* isolate. Data points represent the means ± SD (n = 4).

The enzyme activity is greatly affected by pH because substrate binding and catalysis are dependent on the charge distribution of both the substrate and the enzyme molecules. The reaction pH was adjusted to 4.0–10.0 with various buffers as described above. The optimum pH of *T. harzianum* xylanase is pH 6.0 with an activity of 40 U/ml (Fig. 8a). The enzyme is fairly stable at pH 6.0 and remains active (Fig. 9) retaining > 70% of its activity over 4 h. Souza et al.^64^ reported that the xylanase from *Thermoascus aurantiacus* expressed in *E. coli* showed optimum activity and stability at a similar pH. Yadav et al.^29^ reported that xylanase from *Anoxybacillus kamchatkensis* NASTPD13 showed high activity between pH 6.0 to 9.0 and at pH 6.0, the enzyme retained 71% of its activity over 24 h. The purified 60–79% ammonium sulphate fraction was further confirmed to contain the same protein as that purified in the 50% fraction as it displayed the same pH optimum and size (pH 6.0) (Fig. 8a). Thus, the purified fractions of the 50–79% ammonium sulphate fractions can be combined to increase the yield (%).

#### Optimum temperature and thermal stability

The experiment was carried out at different reaction temperatures ranging from 40 to 79 °C to find the optimal temperature of the xylanase. The highest activity of xylanase was observed at 65 °C (Fig. 8b). Thermal stability data illustrated in Fig. 9 shows that the enzyme retained > 70% activity at 65 °C for 4 h. A similar result was reported by de Oliveira Simões et al.^65^. However, in that study, the enzyme was subjected to treatment for 24 h and was stable for 1 h. The purified 60–79% ammonium sulphate fraction contained the same purified protein as the 50% fraction, with the same molecular weight, pH and temperature optima obtained (65 °C) (Fig. 8b). This confirms that these fractions are not isoforms of the xylanase produced. However, the shape of the curve for the 50% ammonium sulphate fraction is different from the other fractions, which seem to show an optimum rather than a broad bell shape.

The advantages of enzymes that prefer high temperatures are well known because the solubility of the reagents and products is increased, the viscosity is reduced, and the mass transfer rate is higher^66^. When looking for enzymes for industrial uses, stability, and activity at high temperatures are highly desirable.

#### Effect of metal ions and inhibitors

The effects of 8 metal ions (Ca^2+^, Co^2+^, Fe^2+^, Mg^2+^, Mn^2+^, Zn^2+^, K^+^, and Na^+^) at a final concentration of 2 mM and 10 mM on xylanase activity were determined (Table 9) at the optimal pH and temperature (6.0 and 65 °C). Enzyme activity was slightly increased by 2 mM Mn2^+^, K^+^, and Na^+^ (101.11–101.77 U/ml) whereas the enzyme activity was slightly but significantly higher with 10 mM Ca^2+^, Co^2+^, Fe^2+^, Mg^2+^, Zn^2+^ (104.27–110.89 U/ml) (p ≥ 0.05) and thus, these ions act as cofactors for the enzyme. Maximum enhancement was observed for Fe^2+^ (10.88%) followed by Mg^2+^ (9.43%) and Zn^2+^ (8.43%) at 10 mM. Fu et al.^43^ reported similar findings for xylanase from *Trichoderma* sp. TPS-36.

**Table 9:** Effect of metal ions on purified xylanase activity (relative activity %).

	Concentration (mM)	
Metal ions	2.0	10.0
None	100	100
CaCl	84.71	104.27
CoCl_2_	100.20	104.59
FeSO_4_	97.62	110.89
MgSO_4_	96.56	109.44
MnSO_4_	101.77	100.96
ZnSO_4_	94.05	108.55
KH_2_PO_4_	101.98	110.91
NaCl	101.11	98.76

Inhibitory effects were observed for Fe^2+^ (15.29%), Mg^2+^ (3.44%), Zn^2+^ (5.95%) at 2 mM and Na^+^ (1.24%) at 10 mM, however, this inhibition of xylanase was weak (< 50%). Co^2+^ and Ca^2+^ had no effect on xylanase activity (100%) at either concentrations.

Fu et al*.*^43^ also reported weak (< 50%) inhibition of xylanase with the same ions and that Co^2+^ and Ca^2+^ had no effect on xylanase activity (100%) at either concentrations.

#### Substrate specificity of purified xylanase

To determine the substrate specificity of the xylanase for polysaccharide degradation, potential substrates, including birchwood xylan, beechwood xylan, wheat arabinoxylan (soluble and insoluble), xylan from Larchwood, CMC and Avicel were tested under optimal conditions (pH 6.0 and temperature 65 °C) using the purified xylanase. Higher hydrolytic activity was observed for the xylans from beechwood, birchwood, and Larchwood compared to wheat arabinoxylan (Table 10). The xylanase most actively degraded birchwood xylan (174.07%), followed by Larchwood xylan (131.03%), and presented the lowest activity towards wheat arabinoxylan (soluble 70.54% and insoluble 46.62%). The purified xylanase exclusively hydrolyzed xylans, with no activity on CMC and Avicel. This suggested that xylanase's substrate-binding domain has a high affinity for xylans from softwood (birchwood and beechwood)^67^. This might be due to differences in xylan polymer structures and the presence of reactive groups on the surface that are more readily bound. The purified xylanase exhibited significant hydrolytic activity on the diverse xylan substrates, indicating that it might be classified as an endo-1,4-xylanase^68^.

**Table 10:** Substrate specificity of the purified xylanase.

Substrates	Relative activity
Beechwood xylan	100
Birchwood xylan	174.07
Xylan from Larchwood	131.03
Wheat arabinoxylan (soluble)	70.54
Wheat arabinoxylan (Insoluble)	46.62
CMC	ND
Avicel	ND

Each data point represents mean ± SD (n = 3) ND is not detected.

#### Kinetic analysis

**Figure 10:**
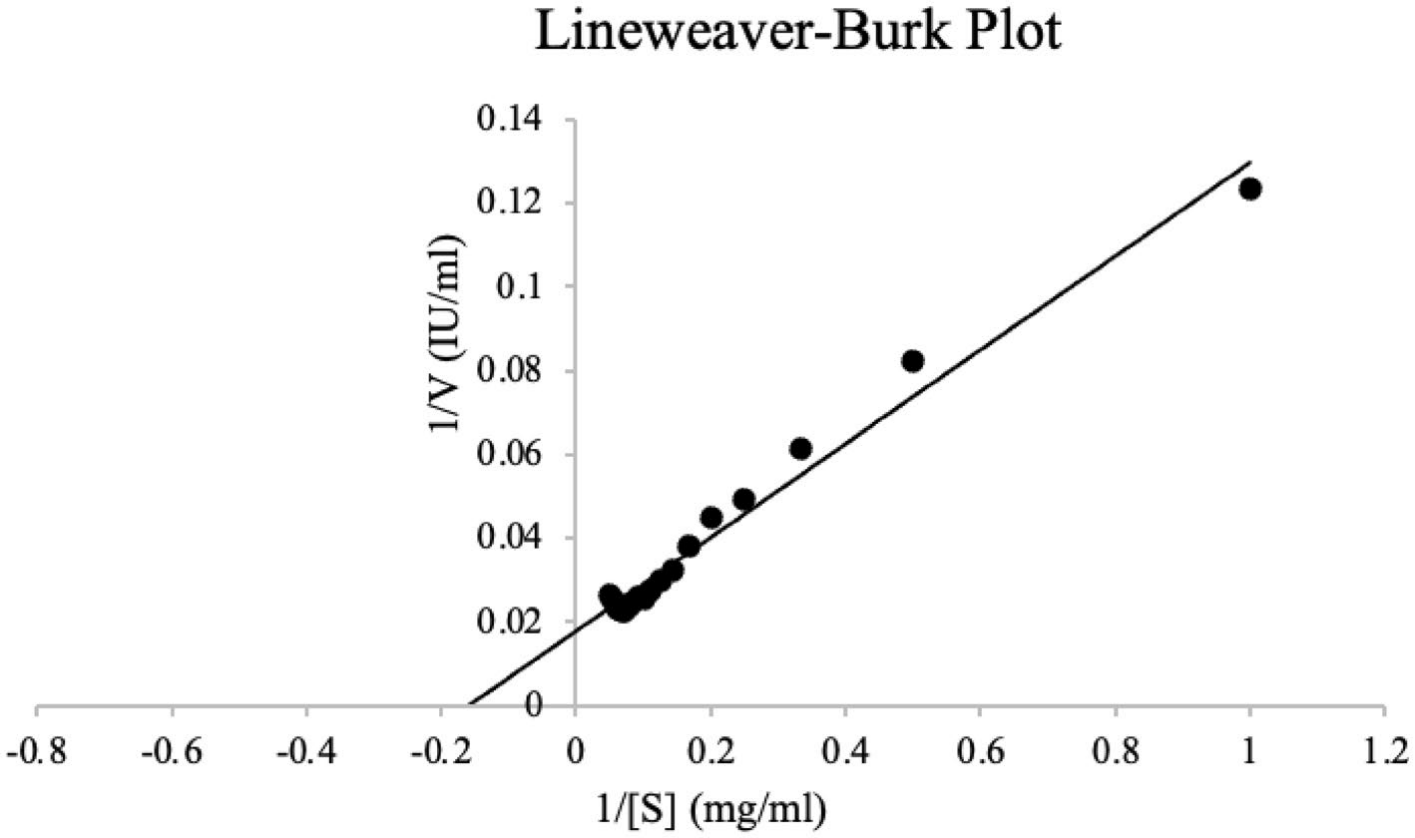
Double reciprocal plot of the purified (50% ammonium sulphate fraction) xylanase from *Trichoderma harzianum* on beechwood xylan. Data points represent the means ± SD (n = 3).

The Michaelis constant, *K*_*m*_, may be determined by measuring the substrate concentration at half the maximum velocity. *K*_*m*_ is a constant that remains fixed for every given enzyme and substrate combination. As a result, a low *K*_*m*_ improves the enzyme's affinity for the substrate^69^ The concentration range of the substrate under investigation was 1–20 mg/ml, the study revealed *K*_*m*_ and *V*_*max*_ were 5.56 mg/ml and 1052.63 µmol/min/mg (Fig. 10). The value of *K*_*m*_ is within the range of fungal xylanases reported in literature (0.14–14 mg/ml). Raj et al.^70^ obtained similar values (4.96 mg/ml and lower *V*_*max*_ 402 µmol/mg/ml) for xylanase from alkaliphilic *Bacillus licheniformis*. Fu et al.^43^ reported high *V*_*max*_ (1250 µmol/min/mg) similar to this study. Because xylanase has a high *V*_*max*_ value and a low *K*_*m*_ value, it has a high affinity for the substrate, beechwood xylan, and can catalyze it more efficiently and quickly than other substrates. Xylanases from *Caldicoprobacter algeriensis* sp. nov. strain TH7C1T were shown to have high selectivity for beechwood xylan^42^.

## Conclusion

The current study describes the successful optimization of xylanase production via statistical modelling using PBD and BBD by a *T. harzianum* strain in submerged fermentation. The most influential independent variables were identified and optimized—resulting in a 4.16-fold and 2.24-fold increase in enzyme activity with BBD compared to the OFAT and PBD, respectively. PBD allowed for the consideration of various variables and avoided loss of information, which might be essential in the optimization of the fermentation process. The predictions of the mathematical models were validated by experimental results. Quadratic models with three independent variables were shown to accurately define xylanase production, with high *R*^2^ values for correlations between the actual and predicted values of the response variables. Results showed high enzyme activities obtained within a high pH range which indicates the potential of the xylanase for use over a wide range of applications. Acidic-thermostable xylanase was purified with a 10.42% recovery and 2.52-fold purity. The specific activity of purified xylanase was 254.63 U/mg. The acidic-thermostability of *T. harzianum* xylanase is advantageous for animal feed manufacturing. Future studies will include scaling up the production of xylanase from *T. harzianum* under optimized conditions, which include the factors and their variables that resulted in the highest xylanase activity (RSM, run 8). Studies will also include sequencing the xylanase protein to understand the structure-guided function of this enzyme.

## Supplementary Information

Supplementary Figures.

## Data Availability

The datasets used and/or analysed during the current study are available from the corresponding author upon reasonable request. Other data generated or analysed during this study are included in this article [and its supplementary information file]**.**
