# Supplementary material for: Optimization, purification, and characterization of xylanase production by a newly isolated Trichoderma harzianum strain by a two-step statistical experimental design strategy
Source: Sci Rep. 2022 Oct 22;12:17791. doi: 10.1038/s41598-022-22723-x (PMC9588001; doi:10.1038/s41598-022-22723-x)
Supplement: Supplementary file 1 — Supplementary Figures. [file 41598_2022_22723_MOESM1_ESM.pdf]

# Optimization, Purification, and Characterization of Xylanase Production by a Newly Isolated *Trichoderma harzianum* strain by a Two-Step Statistical Experimental Design Strategy

**Priyashini Dhaver[a*], Brett Pletschke[b], Bruce Sithole[c,d], Roshini Govinden[a]**

[a]Discipline of Microbiology, School of Life Sciences, Westville Campus, University of KwaZulu-Natal, Durban-4000, South Africa, Email- govindenr@ukzn.ac.za; Tel: 0312608281

[b]Enzyme Science Programme (ESP), Department of Biochemistry, Microbiology, Rhodes University, Makhanda (Grahamstown), Eastern Cape, South Africa

[c]Biorefinery Industry Development Facility, Council for Scientific and Industrial Research, Durban 4000, South Africa

[d]Discipline of Chemical Engineering, University of KwaZulu-Natal, Durban 4000, South Africa

*pdhaver10@gmail.com

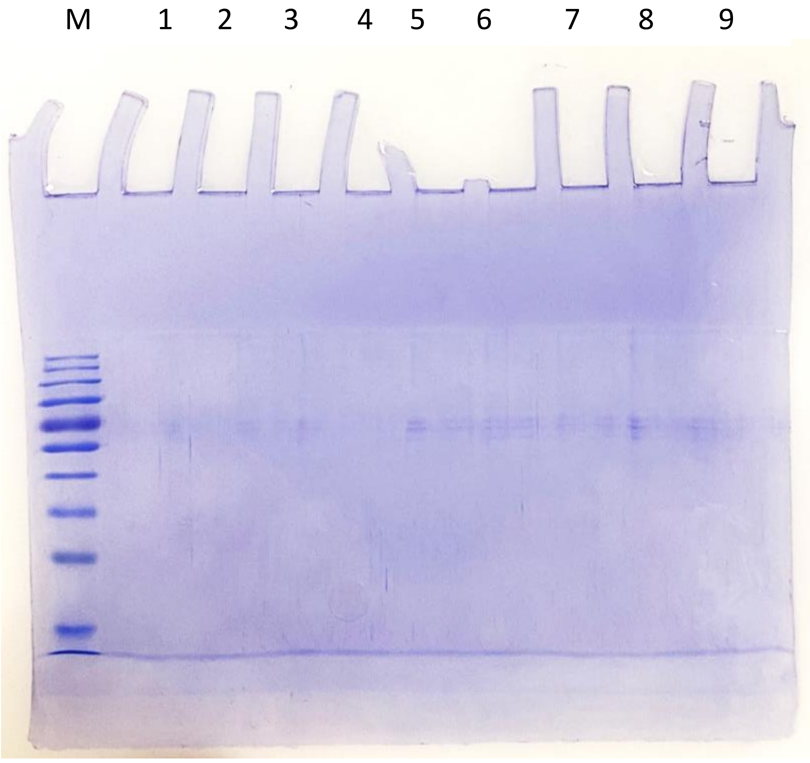


Supplementary Figure 1: Original 12% SDS PAGE gel image representing lanes M: Molecular weight marker (Thermoscientific, USA), 1-4: 50, 60, 70 and 80% ammonium sulphate fractions purified (not concentrated) of xylanase production from *Trichoderma harzianum*, and 5-8: 50, 60, 70 and 80% ammonium sulphate fractions purified (concentrated). Lanes M, and 5-8 were cropped for the manuscript and are represented in Figure 5a, Lanes M, and 5-8.

M 1 2 3 4 5 6 7 8

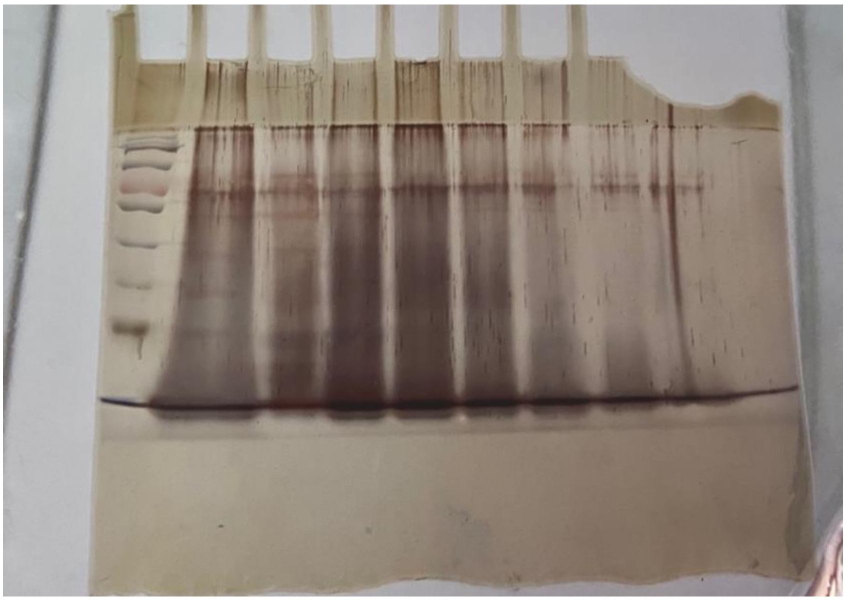

Supplementary Figure 2: Original 12% SDS PAGE gel (silver stained) image representing lanes M: Molecular weight marker (Thermoscientific, USA), 1: crude, 2-8: 30, 40, 50, 60, 70, 80 and 90% ammonium sulphate fractions of xylanase production from *Trichoderma harzianum*. Lanes 4-7 were cropped for the manuscript and are represented in Figure 5a, Lanes 1-4.

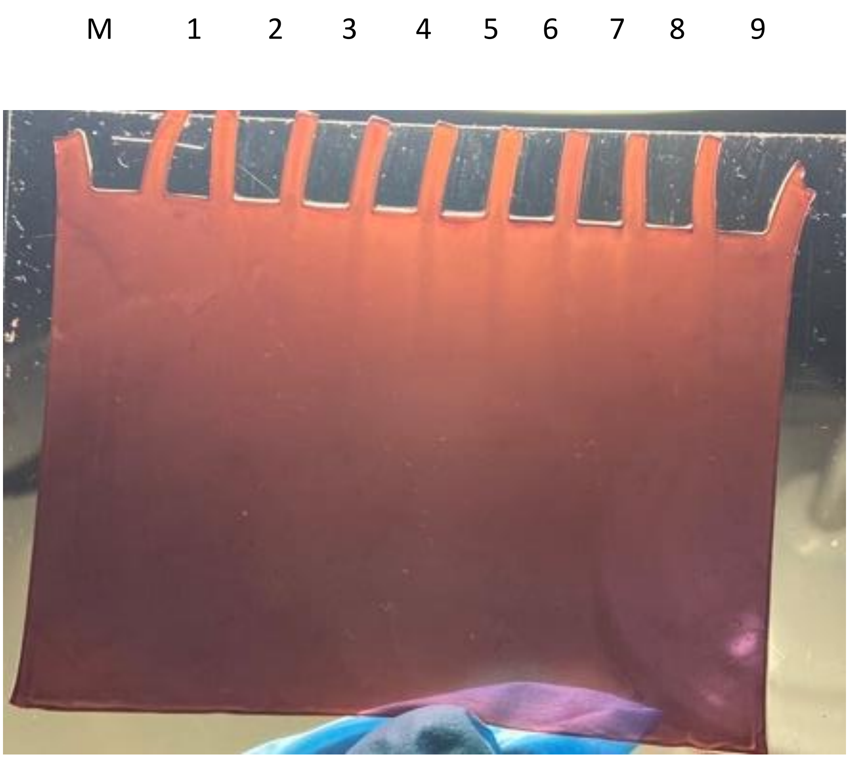


Supplementary Figure 3: Original native-substrate PAGE gel (stained) image representing lanes M: Molecular weight marker (Thermoscientific, USA), 1: crude of xylanase from *Trichoderma harzianum*,, 2-8: 30, 40, 50, 60, 70, 80 and 90% ammonium sulphate fractions showing zones of clearance. Lane 5 was cropped for the manuscript and is represented in Figure 5b, Lane 1.

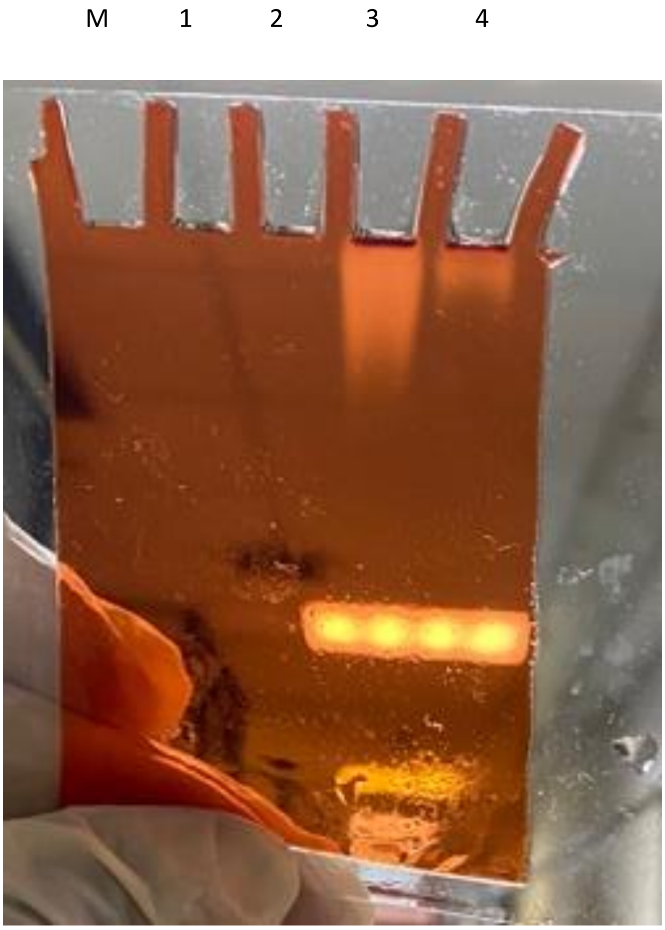


Supplementary Figure 4: Original native-substrate PAGE gel (stained) image representing xylanase production from *Trichoderma harzianum*, lanes M: Molecular weight marker (Thermoscientific, USA), 1: crude, (not concentrated), 2: purified 50% fraction (not concentrated), 3: crude (concentrated), and 4: purified 50% fraction showing zones of clearance. Lane 4 was cropped for the manuscript and is represented in Figure 5b, Lane 2.

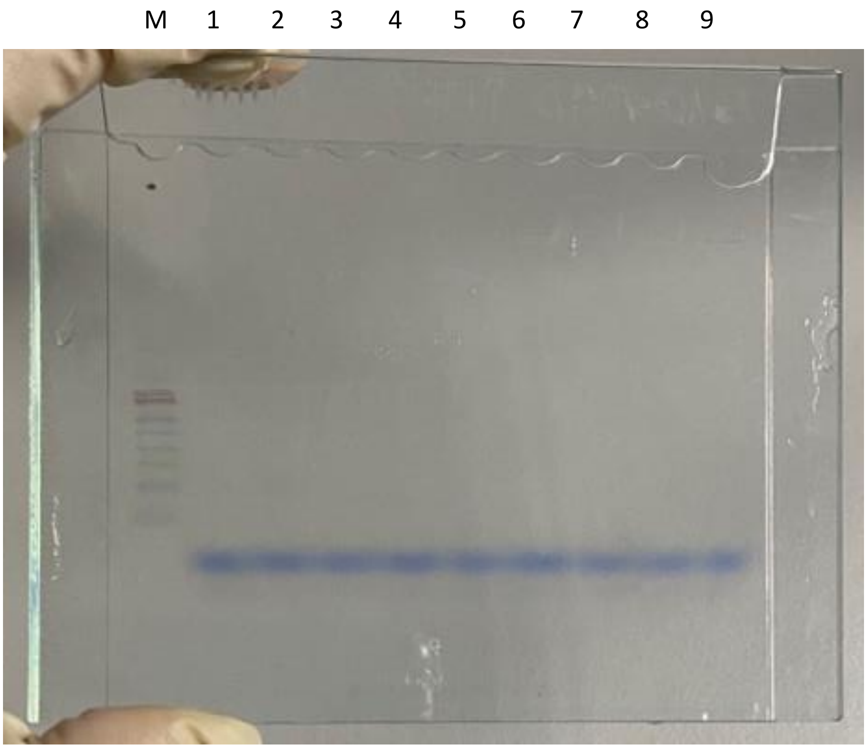


Supplementary Figure 5: Original native-substrate PAGE gel (unstained) image representing xylanase production from *Trichoderma harzianum*, lanes M: Molecular weight marker (Thermoscientific, USA), 1: crude, 2-9: 20, 30, 40, 50, 60, 70, 80 and 90% ammonium sulphate fractions. Lane M was cropped for the manuscript and is represented in Figure 5b, Lane M.

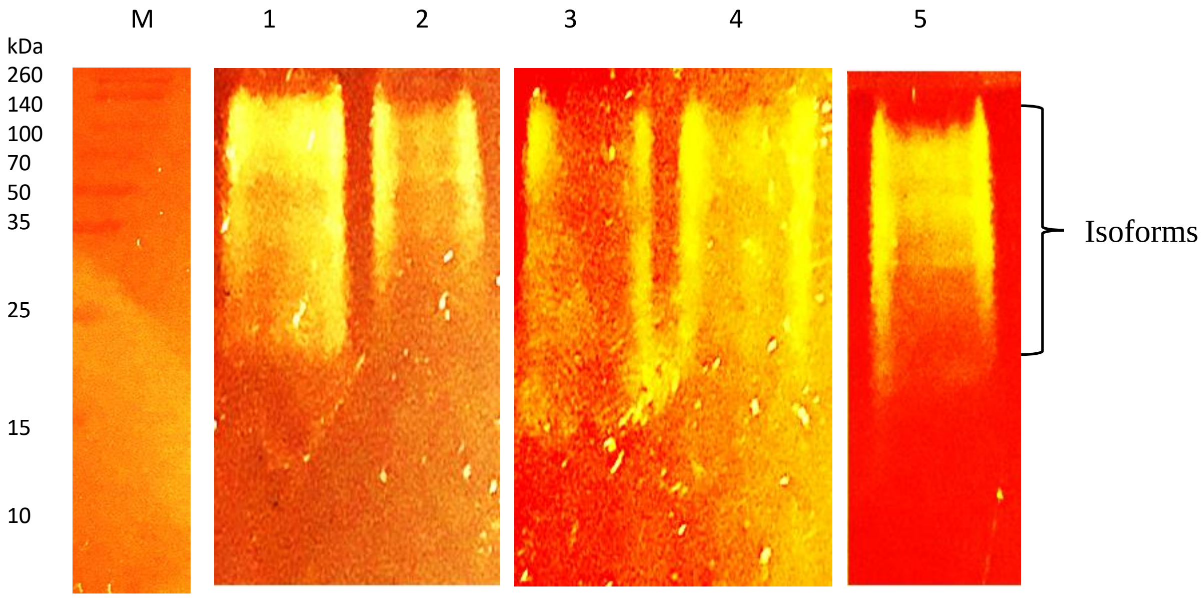


Supplementary Figure 6: Native substrate PAGE gel (1% xylan) of the *Trichoderma harzianum* crude xylanases from the Box Behnken runs 4, 6, 7, 8 and 12 from RSM. lane M: Spectra multicolour broad range marker (Thermo Scientific, USA), lane 1: Box Behnken run 4, lane 2: Box Behnken run 6, lane 3: Box Behnken run 7, lane 4: Box Behnken run 8 and lane 5: Box Behnken run 12.

1 2 3 4 5 6

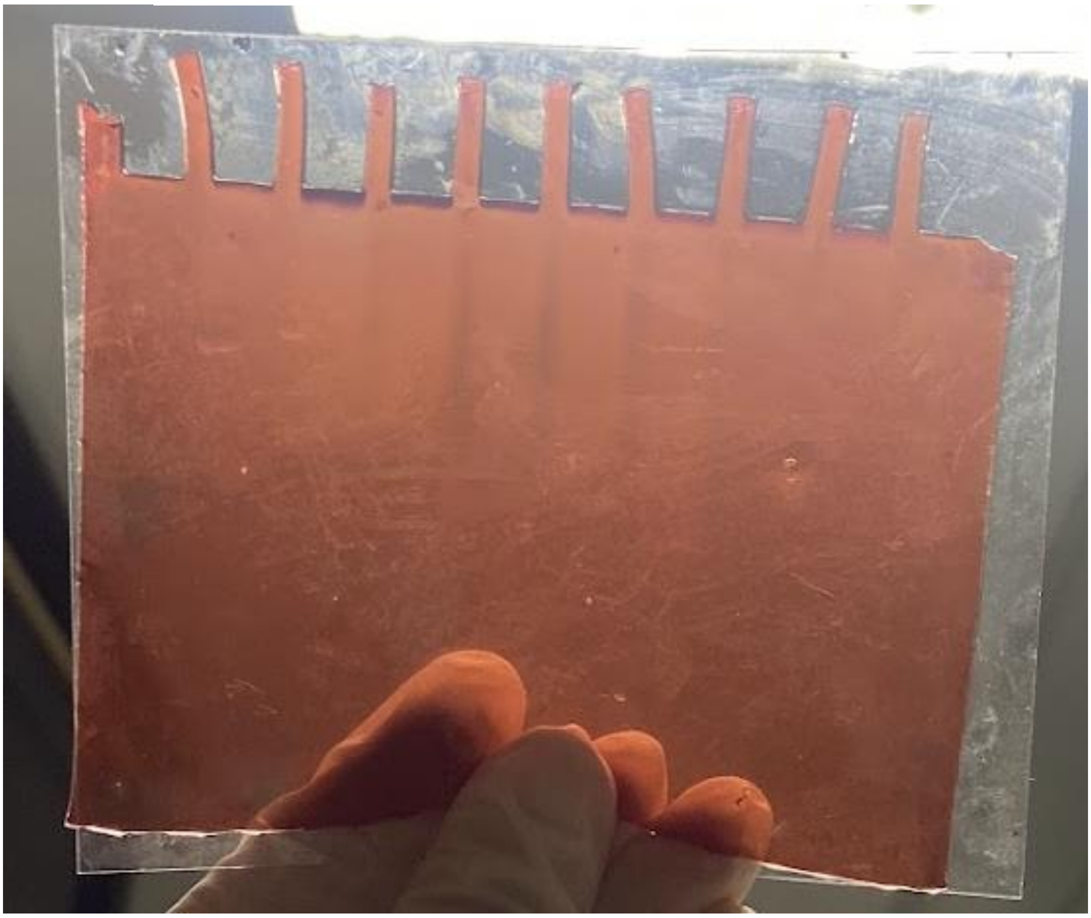

Supplementary Figure 7: Original image of the native substrate PAGE gel (1% xylan) of the *Trichoderma harzianum* crude xylanases from the Box Behnken runs from RSM represented in supplementary Figure 6 . Lanes 2 to 5:  Box Behnken runs 4, 6, 7, and 12 and lane 6: Box Behnken run 12.
